# Modelling of South African Hypertension: Application of Panel Quantile Regression

**DOI:** 10.3390/ijerph19105802

**Published:** 2022-05-10

**Authors:** Anesu Gelfand Kuhudzai, Guido Van Hal, Stefan Van Dongen, Muhammad Ehsanul Hoque

**Affiliations:** 1 Department of Social Epidemiology and Health Policy, University of Antwerp, 2610 Antwerp, Belgium; guido.vanhal@uantwerpen.be; 2 Statistical Consultation Services, University of Johannesburg, Johannesburg 2006, South Africa; 3 Department of Evolutionary Ecology and Biology, University of Antwerp, 2610 Antwerp, Belgium; stefan.vandongen@uantwerp.be; 4 Research Department, Management College of Southern Africa, Durban 4001, South Africa; muhammad.ehsanul@gmail.com

**Keywords:** hypertension, fixed effects model, random effects model, panel quantile regression, South Africa

## Abstract

Hypertension is one of the crucial risk factors for morbidity and mortality around the world, and South Africa has a significant unmet need for hypertension care. This study aims to establish the potential risk factors of hypertension amongst adults in South Africa attributable to high systolic and diastolic blood pressure over time by fitting panel quantile regression models. Data obtained from the South African National Income Dynamics Study (NIDS) Household Surveys carried out from 2008 to 2018 (Wave 1 to Wave 5) was employed to develop both the fixed effects and random effects panel quantile regression models. Age, BMI, gender (males), race, exercises, cigarette consumption, and employment status were significantly associated with either one of the BP measures across all the upper quantiles or at the 75th quantile only. Suggesting that these risk factors have contributed to the exacerbation of uncontrolled hypertension prevalence over time in South Africa.

## 1. Introduction

Hypertension is one of the crucial risk factors for morbidity and mortality around the world [1]. About 7.5 million deaths, which is equivalent to about 12.8% of the total annual deaths globally, occur due to elevated blood pressure [2]. The prevalence of raised blood pressure is estimated to increase to up to about 1.56 billion adults in 2025 unless effective preventive measures are implemented [3].

High blood pressure is defined by a systolic blood pressure ≥140 mmHg and or diastolic blood pressure ≥90 mmHg [2]. Hypertension is known as a silent killer since it is rare for any symptom to be seen especially in its early stages [4]. This asymptomatic and persistent nature of the disease presents a major problem of identifying people with uncontrolled hypertension [5]. High blood pressure symptoms such as headaches, dizziness, nosebleeds, altered vision and fainting episode may manifest when very high levels of systolic blood pressure ≥200 mmHg are experienced [6]. It is only through measurements that detection can be done.

According to Berry et al. (2017), South Africa has a significant unmet need for hypertension care, 91.1% of the hypertensive population was unscreened, undiagnosed, untreated or uncontrolled. The hypertension care cascade revealed that 49% of those with hypertension were lost at the screening stage, 50% of those who were screened never received a diagnosis, 23% of those who were diagnosed did not receive treatment and 48% of those who were treated did not reach the threshold for control [7]. Important efforts are therefore needed to curb the burden of hypertension in South Africa. Modelling of potential risk factors on the upper tail ends of both the diastolic and systolic blood pressure distributions could be ideal in addressing the rising challenge of hypertension in South Africa.

Most studies in South Africa have utilised cross-sectional data and mean regression techniques in an attempt to model determinants of elevated blood pressure [8,9,10,11]. The primary limitation of a cross-sectional study is that possible results and conclusions are based on a short period of time and cannot analyse behaviour of an event over a long period of time [12]. On the other hand, the main loophole of mean regression is utilising the mean across the whole distribution of a response variable [13]. In some cases, the researcher’s interest may not be on the centre of the distribution but rather in its tails [14].

In an attempt to contribute to the hypertension literature and overcome the limitations of engaging the cross-sectional data and mean regression techniques, the aim of this paper is to establish the potential risk factors of hypertension amongst adults in South Africa attributable to high systolic and diastolic blood pressure over time by fitting panel quantile regression models. Panel QR has the capability to identify heterogeneous covariates effects and describe differences in longitudinal changes at different quantiles of the outcome, and provides more robust estimates when heavy tails and outliers exist [15].

## 2. Materials and Methods

The data and variables, panel quantile regression theoretical models and data analysis techniques applied in this paper are presented in this section.

### 2.1. Data and Variables

This was a longitudinal study conducted using data obtained from the South African National Income Dynamics Study (NIDS) Household Surveys carried out from 2008 to 2018 (Wave 1 to Wave 5). At each subsequent wave, new study participants were added to the study to maintain its size and representativeness.

However, the sample of the current study was extracted from individuals who participated in all the five cross-sectional NIDS household surveys carried out in 2008 (Wave 1), 2010–2011 (Wave 2), 2012 (Wave 3), 2014–2015 (Wave 4) and 2017–2018 (Wave 5). Participants gave their consent to participate in the five study waves. After performing data cleaning on the variables used in the current study, 11,362 participated in Wave 1, 13,126 in Wave 2, 16,395 in Wave 3, 18,205 in Wave 4 and 21,180 in Wave 5. A final balanced panel dataset of 3 605 adults aged 18 years and above was extracted from respondents who took part in all the five waves.

A range of socio-demographic and lifestyle variables were selected. These include age, gender, race, BMI, exercises, cigarette consumption, depression and employment status. Blood pressure was measured by systolic blood pressure (SBP) and diastolic blood pressure (DBP).

The heights and weights of the adults were measured by trained field workers, from which the BMI variable was generated. The measurements were done twice for consistency and reliability. BMI and blood pressure classifications for this study were computed according to World Health Organization (WHO) establishments as in Table 1 and Table 2 respectively.

Since the NIDS household surveys were carried out across the nine provinces of South Africa using multi-stage sampling, the data used in this study is nationally representative. Trained interviewers were assigned to collect data on subjects residing in selected households. The ethical approvals to conduct the NIDS household surveys were granted by the University of Cape Town Faculty of Commerce Ethics Committee.

### 2.2. Panel Quantile Regression

Panel data (also known as longitudinal data) is a dataset that consists of repeated measurements of variables observed on a set of entities or units. The entities or units could be individuals, companies, countries, etc.

Panel data is structured in a vector of the dependent variable $y_{\left[n\right]}^{t}$ observed on *n* units and a matrix of *p* independent variables $X_{\left[nxp\right]}^{t}$ where *t* = 1, …, *T* is the number of times [16]. Panel data can either be balanced or unbalanced. Balanced panel data occurs when each case is observed for each time occasion and unbalanced when different number of occasions are observed for each case.

Longitudinal data can be analysed by either the fixed or random effects models. According to Davino et al. (2014) a fixed model can be expressed as:

(1) $$y^{t}=\alpha +\beta x^{t}+e^{t}\;$$

where α is a vector of the unknown intercept for each unit.

$e^{t}$ is the error term.

A fixed panel model aims to remove the unit time invariant characteristics and to analyse the predictors’ net effect. Therefore, the α measures the unobserved heterogeneity.

A quantile regression model for the analysis of panel data with fixed effects [17] is given by:

(2) $$Q_{\theta \;}\left(y^{t}|x\right)=\alpha +\beta \left(\theta \right)x^{t}+e^{t}\;$$

where θ represents the vector of quantiles.

The random model can be expressed [16] as:

(3) $$y^{t}=\alpha +\beta x^{t}+\epsilon +e^{t}\;$$

where α is the classical average effect.

ϵ is the random deviation of unit intercepts from α.

A quantile regression model for the analysis of panel data with random effects [18] is given by:

(4) $$Q_{\theta \;}\left(y^{t}|x\right)=\alpha +\beta \left(\theta \right)x^{t}+\epsilon +e^{t}$$

The quantile regression model for the analysis of panel data with random effects aims to controls for time-invariant dependence between the fixed effects and a set of covariates [18]. Random effect models are highly recommended for analysing clustered data [19,20].

### 2.3. Data Analysis

Descriptive statistics were used in the study to report the prevalence of hypertension among South African adults by demographic and lifestyle characteristics from year 2008 to 2018 using IBM Statistical Package for the Social Sciences (SPSS) version 28. The panel quantile regression models were fitted using rqpd R package [17]. Thus, both the fixed effects and random effects models.

## 3. Results

This section presents the empirical results of the study in form of tables.

Table 3 and Table 4 illustrate the longitudinal trend (Wave 1 to Wave 5) in prevalence of hypertension attributable to high systolic blood pressure (140 mmHg and above) and diastolic blood pressure (90 mmHg and above) respectively among South African adults by demographic and lifestyle characteristics. Figures S1–S16 (Supplementary Materials) present the visual longitudinal trend (Wave 1 to Wave 5) in uncontrolled hypertension for each demographic or lifestyle characteristic predictor variable.

Coloured participants had the highest prevalence of raised blood pressure across all waves attributable to excessive values of both BP measures ranging from 28.7% to 37.4%. Asian and Indian respondents had the least rates of hypertension over the study time period ranging from 6.7% to 20.0%.

Elevated blood pressure increased with age athwart all waves assignable to high values of both SBP and DBP. This age-specific prevalence of hypertension ranged from 5.2% on 18 to 29 years age group to 48.0% on the 50 years and above age group over the study period. A similar trend emerged with BMI, indicating that elevated blood pressure increased with the level of BMI ranging from 12.5% in underweight to 46.8% in morbidly obese participants.

High blood pressure proportions revealed in Table 3 and Table 4 suggest that respondents who do not participate in physical exercises are more vulnerable to suffering from hypertension between wave 1 and wave 5 explicable to both BP measures. Participants who smoke had higher rates of hypertension (21.2% to 30.1%), as did with those who suffer from depression (17.8% to 28.7%).

Mixed proportions of elevated blood pressure were recorded in regard to gender and employment status ascribable to both high SBP and DBP values. From wave 1 (2008) to wave 5 (2018), the hypertension prevalence attributable to both high values of SBP and DBP among men and women ranges between 18.2% and 28.0%, unemployed participants (19.7% to 27.1%) and employed participants (14.9% to 27.1%).

Table 5 shows the upper panel quantile regression estimated coefficients for SBP’s risk factors obtained using both the fixed effects and random effects approaches. It is apparent from Table 5 that age, BMI and race had positive statistically significant effects on SBP across the estimated upper quantiles (τϵ{0.75,0.95}. Also, in all upper quantiles, gender and employment status presented negative significant impact on SBP. Cigarette consumption was only statistically significant at the 75th quantile. Exercises and depression did not present any statistically significant relations with SBP athwart all quantiles.

Table 6 illustrates the upper longitudinal quantile regression estimated coefficients for DBP’s risk factors derived from applying both the fixed effects and random effects methods. Age, BMI, gender and cigarette consumption displayed statistically significant associations with DBP across all the higher quantiles estimated. Race and depression had statistically insignificant relations with DBP. Exercises and employment status were only significant at the 75th quantile.

## 4. Discussion

South Africa has a significant unmet need for hypertension care [7]. While several studies in South Africa have utilised cross-sectional data and mean regression techniques in an attempt to model determinants of elevated blood pressure, this study employed longitudinal data to fit panel quantile regression models.

It can be seen from this study that hypertension remains a significant public health issue in South Africa since 2008. From the descriptive statistical analysis, males, coloured participants, aged respondents, participants with excessive level of BMI, sedentary respondents, those who smoke and suffer from depression recorded high hypertension prevalence across all waves. These findings were further confirmed by the panel quantile regression analysis results.

Both the fixed effects and random effects panel quantile regression approaches revealed that age, BMI, gender (males), race, exercises, cigarette consumption and employment status were significantly associated with either one of the BP measures across all the upper quantiles or at the 75th quantile only. This is revealing that these risk factors have contributed to the exacerbation of uncontrolled hypertension prevalence over time in South Africa.

The impact of age increase, overweight, obesity and lack of physical exercise participation in exacerbating the risk of uncontrolled hypertension is consistent with earlier studies which suggest that hypertension is common in developing countries caused by ageing of population, bad dietary habits and sedentary lifestyle [21]. Coloured participants had the highest prevalence of raised blood pressure across all waves accountable to excessive values of both BP measures, a finding coherent with an earlier study by [22] which observed that South Africans who are identified as coloured were more likely to be hypertensive than other races in South Africa.

Males were found to be more prone to suffer from uncontrolled hypertension in panel quantile regression, a finding in agreement with a previous study by [4] which also reported that higher odds of being hypertensive were found in male subjects. Similar findings on cigarette consumption or smoking being a risk factor for high blood pressure have been presented in various earlier studies [10,11,23].

Employed participants were found to be more vulnerable to hypertension due to high diastolic blood pressure. This outcome is consistent with the results of a study held in Japan which revealed job strain to be significantly related to hypertension, particularly in the subordinate groups [24].

## 5. Conclusions

This study sought to establish the potential risk factors of hypertension amongst adults in South Africa attributable to high systolic and diastolic blood pressure over time by fitting panel quantile regression models. Applying both the fixed effects and random effects panel quantile regression approaches revealed that age, BMI, gender (males), race, exercises, cigarette consumption and employment status were significantly associated with either of the BP measures across all the upper quantiles or at the 75th quantile only. Suggesting that these risk factors have contributed to the exacerbation of uncontrolled hypertension prevalence over time. Thus, from Wave 1 (2008) to Wave 5 (2017–2018), the estimated regression coefficients from both the fixed effects and random effects panel quantile regression methods were similar, despite literature suggesting that the fixed panel model aims to remove the unit time invariant characteristics, and the random effects aims to control for time-invariant dependence between the fixed effects.

## Figures and Tables

**Table 1:** Body Mass Index Classification.

BMI, kg/m^2^	Classification	Risk of Comorbidities
<18.50	Under weight	Low (But risk of other clinical problems increased)
18.50–24.99	Normal weight	Average
25.00–29.99	Overweight	Increased
30.00–34.99	Class I obesity	Moderate
35.00–39.99	Class II obesity	Severe
≥40.00	Class III obesity	Very Severe

Ref. [2].

**Table 2:** Hypertension Classification.

Blood Pressure Category	Systolic (mmHg)		Diastolic (mmHg)
Normal	Less than 120	AND	Less than 80
Prehypertension	120–139	Or	80–89
Stage 1 Hypertension	140–159	Or	90–99
Stage 2 Hypertension	160 or higher	Or	100 or higher
Hypertensive Crisis (Emergency Care Needed)	Higher than 180	Or	Higher than 110

Ref. [2].

**Table 3:** Hypertension Prevalence among South African Adults by Demographic and Lifestyle Characteristics based on SBP.

SBP (*n* = 3605)						
Waves		Wave 1	Wave 2	Wave 3	Wave 4	Wave 5
		Hypertension	Hypertension	Hypertension	Hypertension	Hypertension
Gender	Male	227 (20.7%)	214 (19.6%)	240 (22.0%)	244 (22.2%)	236 (21.7%)
	Female	493 (20.2%)	499 (20.3%)	495 (20.2%)	446 (18.2%)	461 (18.9%)
Race	African	591 (19.2%)	575 (18.6%)	602 (19.6%)	537 (17.4%)	548 (17.9%
	Coloured	120 (28.7%	126 (30.7%)	126 (30.4%)	144 (34.0%)	138 (33.4%)
	Asian/Indian	2 (14.3%)	2 (13.3%)	1 (7.1%)	3 (20.0%)	1 (7.1%)
	White	7 (22.6%)	10 (30.3%)	6 (18.2%)	6 (18.8%)	10 (30.3%)
Age	18–29 years	79 (6.1%)	65 (5.8%)	57 (5.9%)	40 (5.7%)	22 (5.2%)
	30–39 years	92 (11.8%)	92 (11.8%)	68 (8.5%)	67 (7.8%)	86 (9.2%)
	40–49 years	170 (25.4%)	163 (23.0%)	164 (22.4%)	130 (17.3%)	121 (15.9%)
	50 years & above	379 (48.0%)	393 (42.4%)	446 (42.8%)	453 (36.5%)	468 (33.5%)
BMI	Underweight	30 (12.8%)	22 (12.9%	11 (11.6%)	19 (14.4%)	20 (12.2%)
	Healthy	215 (15.1%	189 (15.0%)	200 (15.8%)	163 (14.2%)	160 (14.1%)
	Overweight	169 (20.8%)	173 (19.3%)	178 (18.7%)	188 (21.5%)	182 (21.0%)
	Obese	161 (28.8%)	161 (24.8%)	167 (25.1%)	163 (22.5%)	157 (22.6%)
	Very Obese	95 (30.5%	98 (28.1%)	117 (31.0%)	102 (24.1%)	97 (24.2%)
	Morbidly Obese	50 (25.8%)	68 (34.0%)	62 (34.1%)	55 (22.6%)	81 (30.6%)
Exercises	Never	564 (21.6%)	580 (21.0%)	576 (21.2%)	584 (20.3%)	555 (20.6%)
	Once or Two times a week	98 (17.7%)	84 (16.3%)	96 (19.0%)	59 (18.8%)	106 (19.3%)
	Three or More times a week	58 (15.5%)	49 (18.0%)	63 (19.5%)	47 (12.9%)	36 (12.7%)
Depression	Rarely or none of the time	333 (19.3%)	405 (19.6%)	361 (19.6%)	366 (19.7%)	378 (20.0%)
	Some or little of the time	244 (21.1%	183 (19.2%)	256 (22.3%)	220 (18.7%)	232 (20.3%)
	Occasionally or All of the time	143 (21.7%)	125 (23.8%)	118 (21.4%)	104 (20.0%)	87 (17.8%)
Cigarette Consumption	No	580 (19.6%)	589 (19.3%)	594 (19.8%)	545 (18.5%)	577 (19.5%)
	Yes	140 (23.9%)	124 (25.2%)	141 (26.1%)	145 (24.0%)	120 (21,2%)
Employment Status	No	536 (19.7%)	553 (21.1%)	523 (21.4%)	505 (21.9%)	498 (21.9%)
	Yes	184 (22.3%)	160 (17.2%)	212 (19.3%)	185 (14.9%)	199 (16.0%)

**Table 4:** Hypertension Prevalence among South African Adults by Demographic and Lifestyle Characteristics based on DBP.

DBP (*n* = 3605)						
Waves		Wave 1	Wave 2	Wave 3	Wave 4	Wave 5
		Hypertension	Hypertension	Hypertension	Hypertension	Hypertension
Gender	Male	234 (21.4%)	230 (21.2%)	278 (25.7%)	249 (23.1%)	272 (25.1%)
	Female	617 (25.6%)	631 (26.3%)	668 (28.0%)	584 (24.1%)	490 (20.4%)
Race	African	714 (23.4%)	711 (23.4%)	799 (26.4%)	672 (22.1%)	627 (20.7%)
	Coloured	130 (31.3%)	140 (34.7%)	141 (35.6%)	154 (37.4%)	128 (31.6%)
	Asian/Indian	2 (13.3%)	1 (6.7%)	1 (7.1%)	2 (13.3%)	3 (20.0%)
	White	5 (16.7%)	9 (29.0%)	5 (15.2%)	5 (16.1%)	4 (12.9%)
Age	18–29 years	119 (9.2%)	118 (10.6%)	129 (13.5%)	75 (10.7%)	49 (11.7%)
	30–39 years	150 (19.5%)	162 (21.0%)	154 (20.0%)	148 (17.6%)	158 (17.0%)
	40–49 years	241 (36.5%)	233 (33.8%)	226 (31.3%)	183 (24.9%)	185 (24.6%)
	50 years & above	341 (43.6%)	348 (38.3%)	437 (43.0%)	427 (35.0%)	370 (26.9%)
BMI	Underweight	37 (16.0%)	28 (16.2%)	14 (14.9%)	20 (15.2%)	20 (12.5%)
	Healthy	233 (16.5%)	205 (16.5%)	260 (20.9%)	187 (16.5%)	182 (16.4%)
	Overweight	202 (24.9%)	221 (24.9%)	220 (23.5%)	220 (25.5%)	191 (22.0%)
	Obese	191 (34.7%)	206 (32.4%)	220 (33.6%)	200 (28.0%)	164 (24.1%)
	Very Obese	116 (38.2%)	119 (34.6%)	151 (41.5%)	122 (29.4%)	117 (29.2%)
	Morbidly Obese	72 (36.9%)	79 (42.0%)	81 (46.8%)	82 (34.6%)	88 (33.5%)
Exercises	Never	672 (26.0%)	686 (25.4%)	745 (28.1%)	700 (24.7%)	600 (22.6%)
	Once or Two times a week	111 (20.3%)	117 (22.9%)	124 (24.8%)	73 (23.8%)	106 (19.6%)
	Three or More times a week	68 (18.2%)	58 (21.6%)	77 (24.4%)	60 (16.8%)	56 (19.6%)
Depression	Rarely or none of the time	368 (21.6%)	483 (23.9%)	477 (26.6%)	432 (23.7%)	413 (22.0%)
	Some or little of the time	298 (25.8%)	243 (25.7%)	321 (28.3%)	276 (23.7%)	256 (22.6%)
	Occasionally or All of the time	185 (28.7%)	135 (26.0%)	148 (27.5%)	125 (24.5%)	93 (19.6%)
Cigarette Consumption	No	710 (24.3%)	740 (24.6%)	787 (26.8%)	679 (23.4%)	618 (21.2%)
	Yes	141 (24.3%)	121 (25.3%)	159 (30.1%)	154 (26.0%)	144 (25.6%)
Employment Status	No	634 (23.5%)	628 (24.5%)	647 (27.1%)	573 (25.2%)	485 (21.5%)
	Yes	217 (26.8%)	233 (25.3%)	299 (27.1%)	259 (21.1%)	277 (22.5%)

**Table 5:** Panel Quantile Regression Estimates for SBP’s Risk Factors.

	Fixed Effects Model		Random Effects Model	
τ	Q (0.75)	Q (0.95)	Q (0.75)	Q (0.95)
Age	0.74 ***	1.15 ***	0.74 ***	1.15 ***
BMI	0.48 ***	0.52 ***	0.48 ***	0.52 ***
Gender	−9.30 ***	−8.59 ***	−9.30 ***	−8.59 ***
Race	3.27 *	3.93 *	3.27 *	3.93 *
Exercises	0.06	−0.33	0.06	−0.33
Cigarette Consumption	0.93 ***	0.79	0.93 ***	0.79
Depression	0.08	0.61	0.08	0.61
Employment Status	−0.89 ***	−2.66 **	−0.89 ***	−2.66 **

* *p*-value < 0.05; ** *p*-value < 0.01; *** *p*-value < 0.001.

**Table 6:** Panel Quantile Regression Estimates for DBP’s Risk Factors.

	Fixed Effects Model		Random Effects Model	
τ	Q (0.75)	Q (0.95)	Q (0.75)	Q (0.95)
Age	2.83 ***	3.80 ***	2.83 ***	3.80 ***
BMI	4.01 ***	3.77 ***	4.01 ***	3.77 ***
Gender	−2.77 ***	−1.38 *	−2.77 ***	−1.38 *
Race	1.38	7.56	1.38	7.56
Exercises	−4.65 *	−6.75	−4.65 *	−6.75
Cigarette Consumption	1.07 ***	1.24 ***	1.07 ***	1.24 ***
Depression	3.07	6.43	3.07	6.43
Employment Status	7.37 ***	−2.61	7.37 **	−2.61

* *p*-value < 0.05; ** *p*-value < 0.01; *** *p*-value < 0.001.

## Supplementary Materials

The following supporting information can be downloaded at: https://www.mdpi.com/article/10.3390/ijerph19105802/s1, Figure S1: Uncontrolled hypertension on Gender based on SBP. Figure S2: Uncontrolled hypertension on Race based on SBP. Figure S3: Uncontrolled hypertension on Age Group based on SBP. Figure S4: Uncontrolled hypertension on BMI based on SBP. Figure S5: Uncontrolled hypertension on Physical Inactive based on SBP. Figure S6: Uncontrolled hypertension on Depressive Participants based on SBP. Figure S7: Uncontrolled hypertension on Cigarette Consumption based on SBP. Figure S8: Uncontrolled hypertension on Employment Status based on SBP. Figure S9: Uncontrolled hypertension on Gender based on DBP. Figure S10: Uncontrolled hypertension on Race based on DBP. Figure S11: Uncontrolled hypertension on Age Group based on DBP. Figure S12: Uncontrolled hypertension on BMI based on DBP. Figure S13: Uncontrolled hypertension on Physical Inactive based on DBP. Figure S14: Uncontrolled hypertension on Depressive Participants based on DBP. Figure S15: Uncontrolled hypertension on Cigarette Consumption based on DBP. Figure S16: Uncontrolled hypertension on Employment Status based on DBP.
