# Supplementary material for: Modelling of South African Hypertension: Application of Panel Quantile Regression
Source: Int J Environ Res Public Health. 2022 May 10;19(10):5802. doi: 10.3390/ijerph19105802 (PMC9141596; doi:10.3390/ijerph19105802)
Supplement: Supplementary file 1 [file ijerph-19-05802-s001.zip › ijerph-1656264-supplementary.pdf]

Supplementary Figures

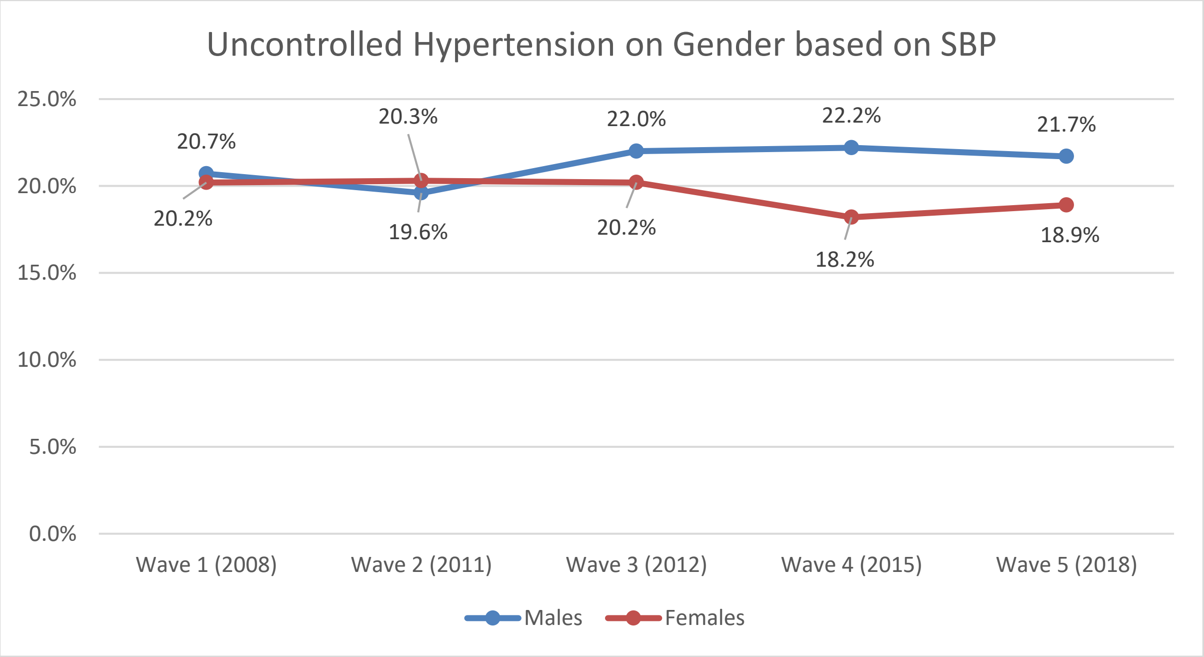

Figure S1: Uncontrolled hypertension on Gender based on SBP

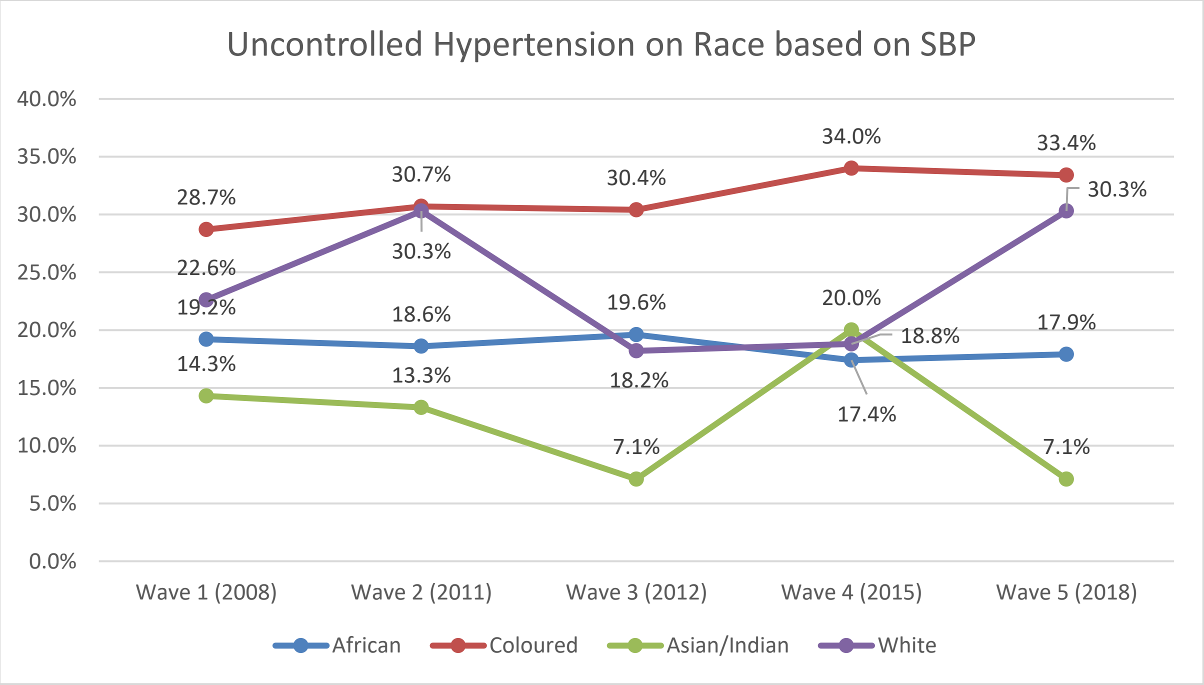

Figure S2: Uncontrolled hypertension on Race based on SBP

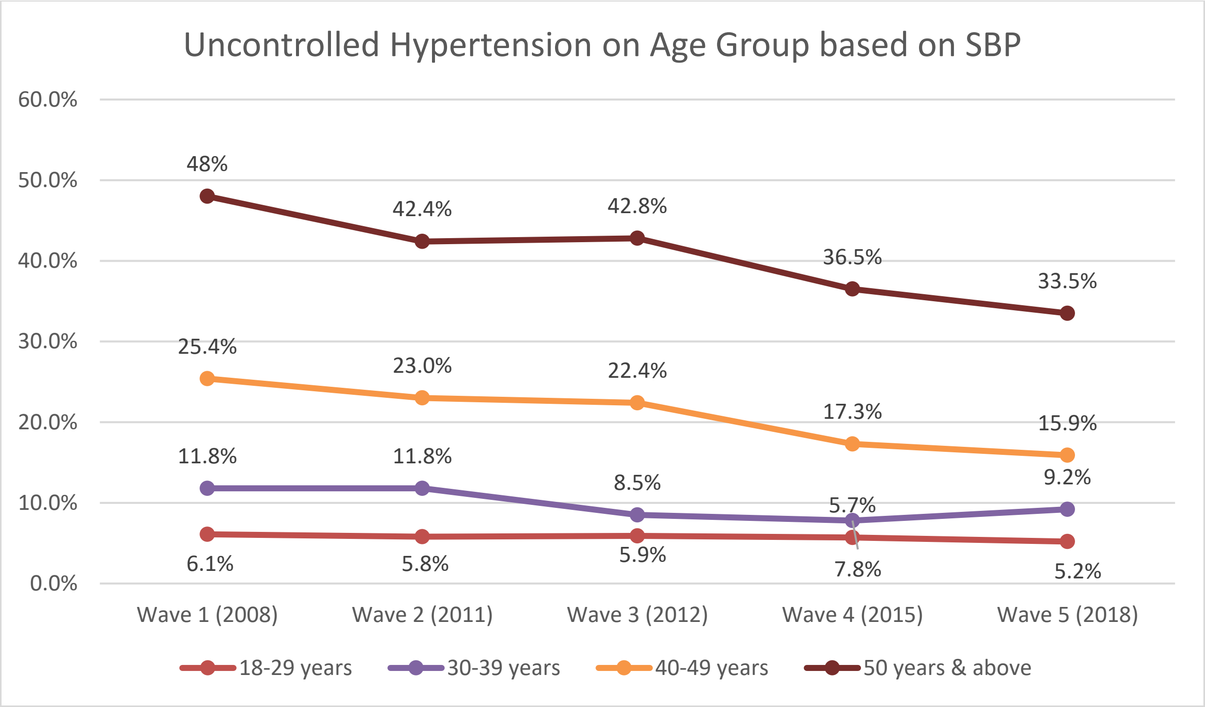

Figure S3: Uncontrolled hypertension on Age Group based on SBP

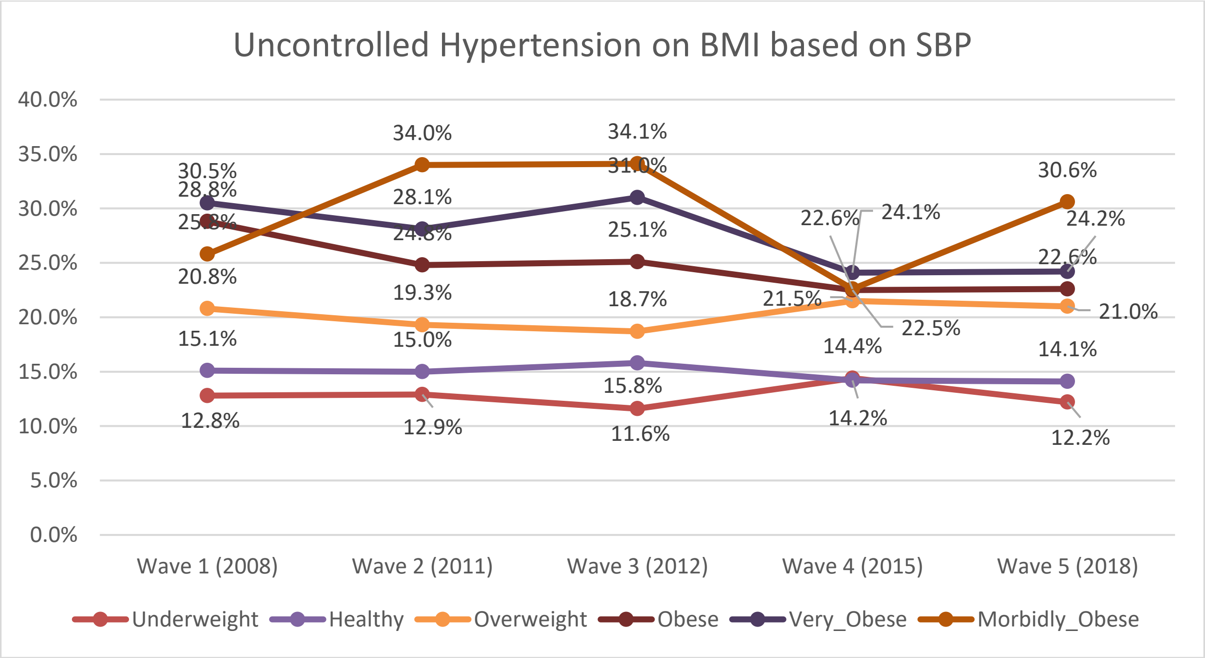

Figure S4: Uncontrolled hypertension on BMI based on SBP

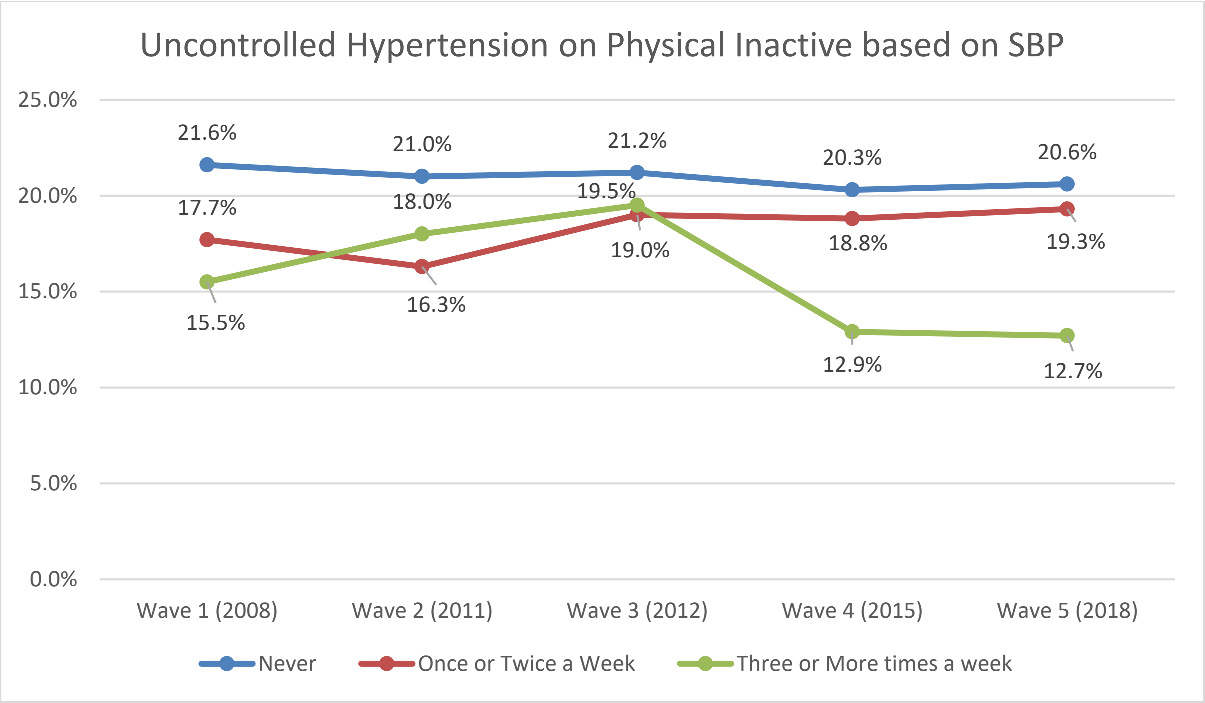

Figure S5: Uncontrolled hypertension on Physical Inactive based on SBP

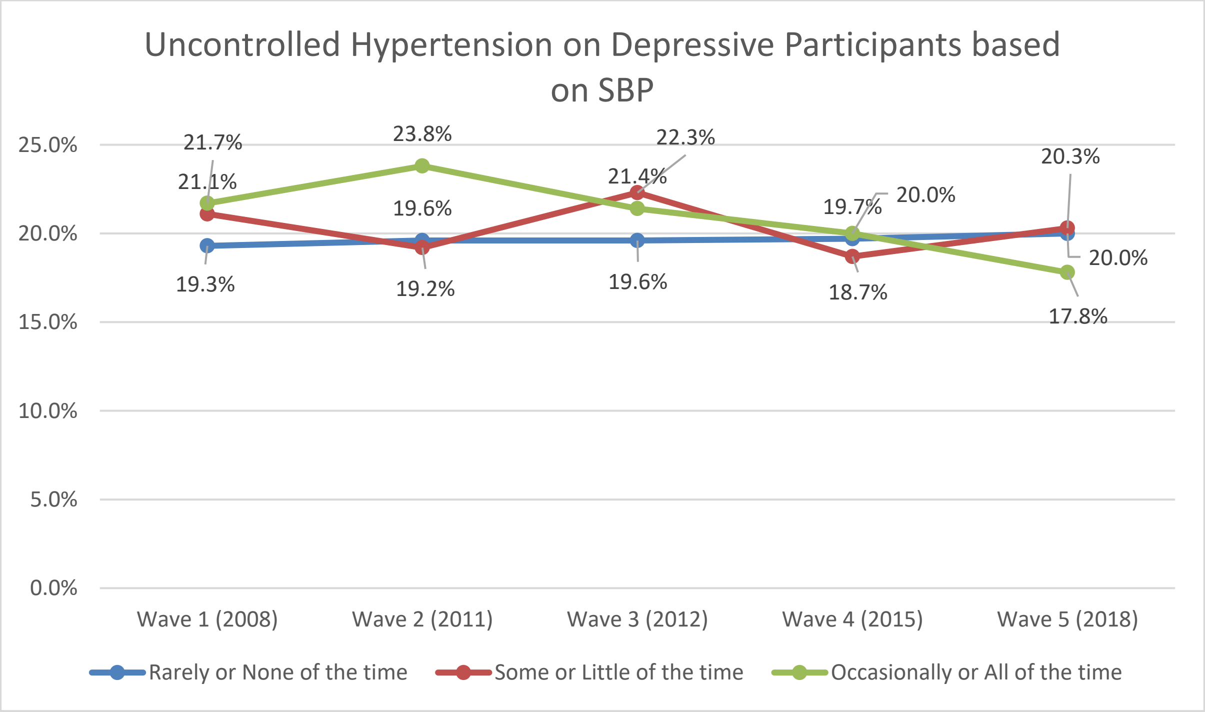

Figure S6: Uncontrolled hypertension on Depressive Participants based on SBP

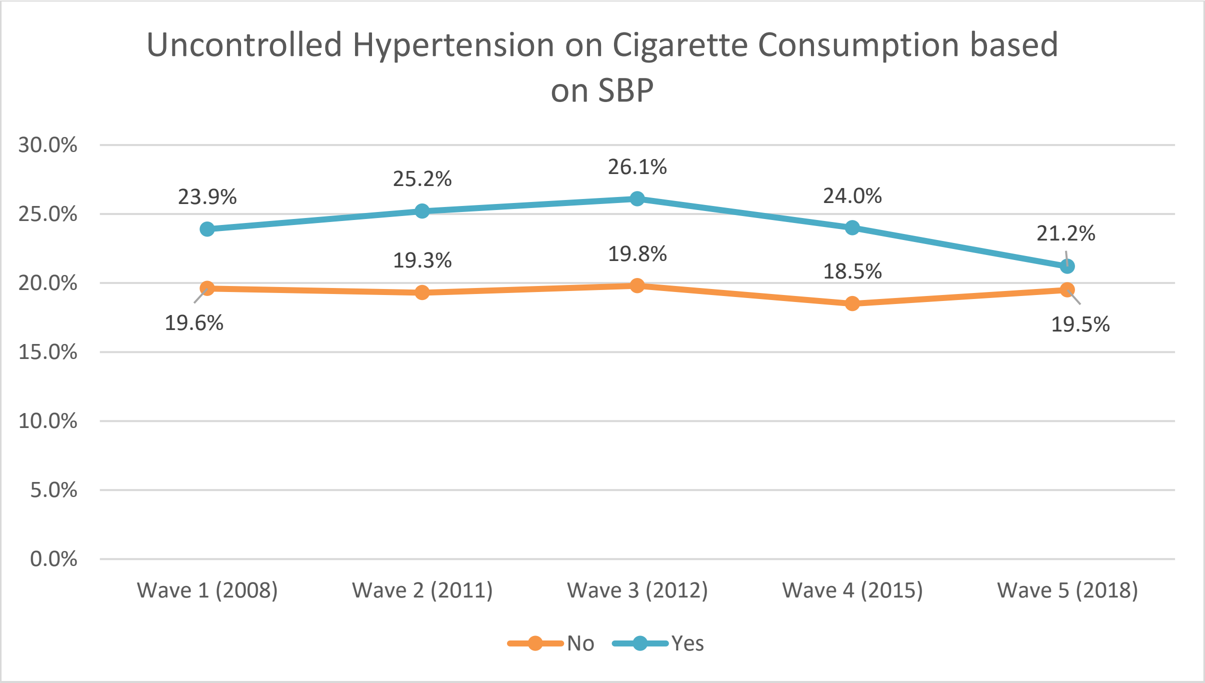

Figure S7: Uncontrolled hypertension on Cigarette Consumption based on SBP

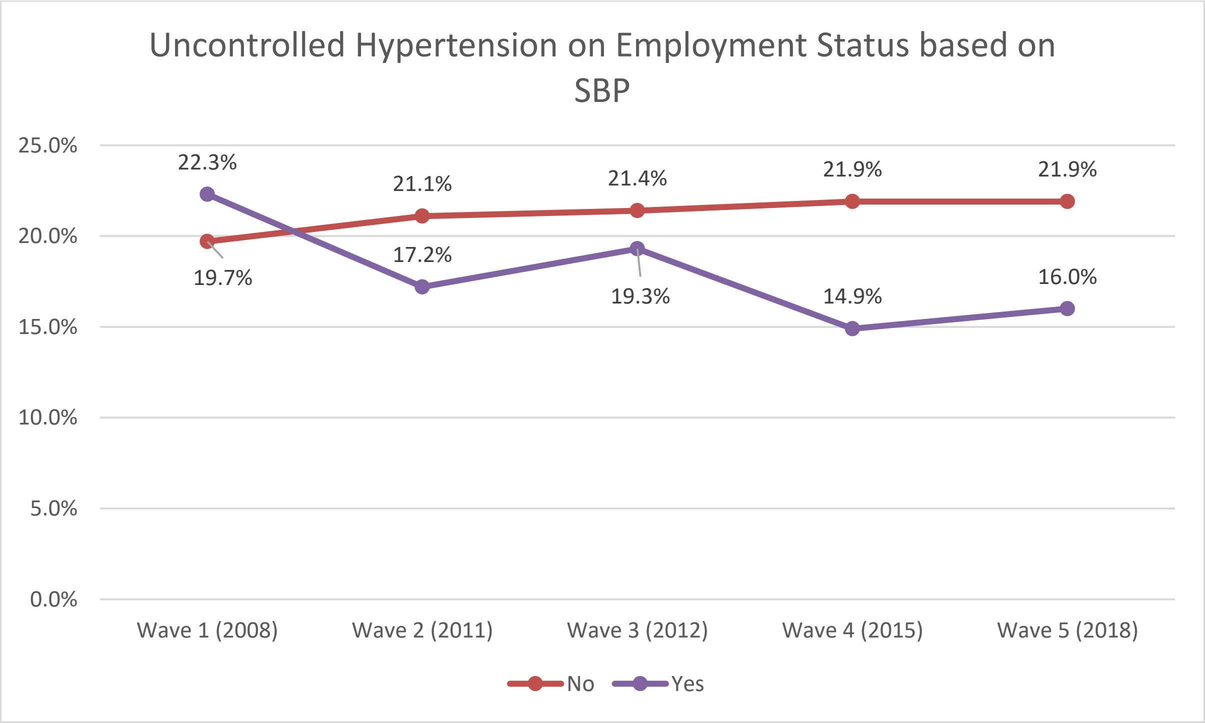

Figure S8: Uncontrolled hypertension on Employment Status based on SBP

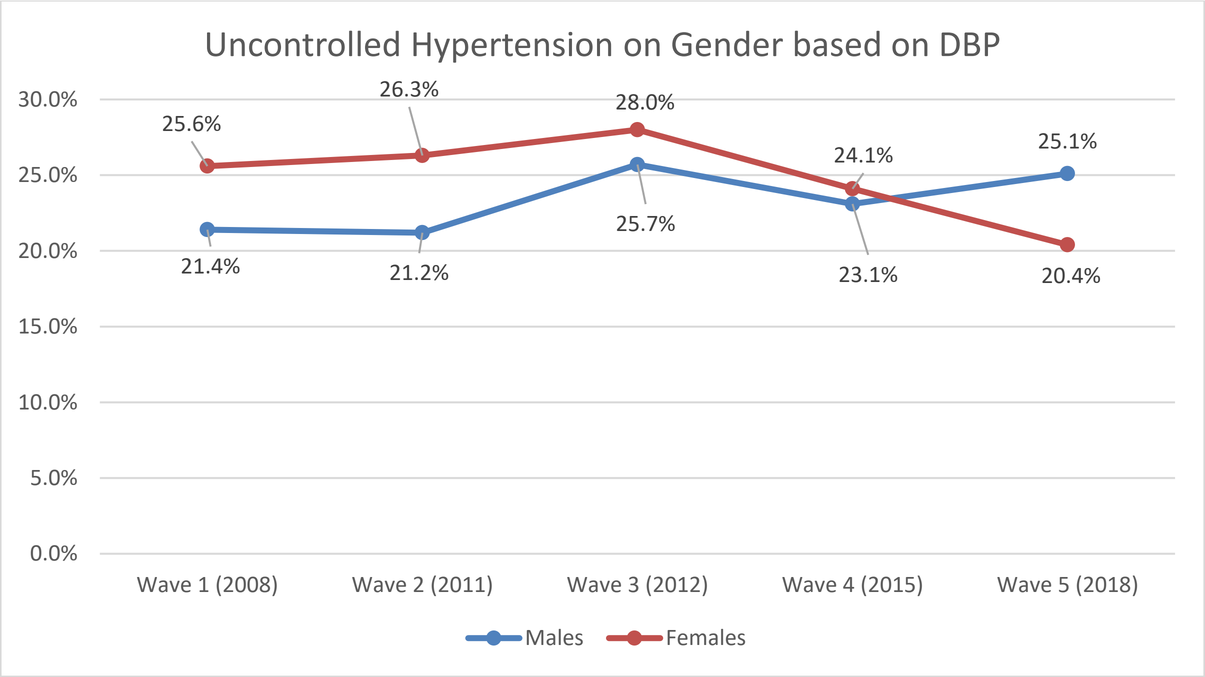

Figure S9: Uncontrolled hypertension on Gender based on DBP

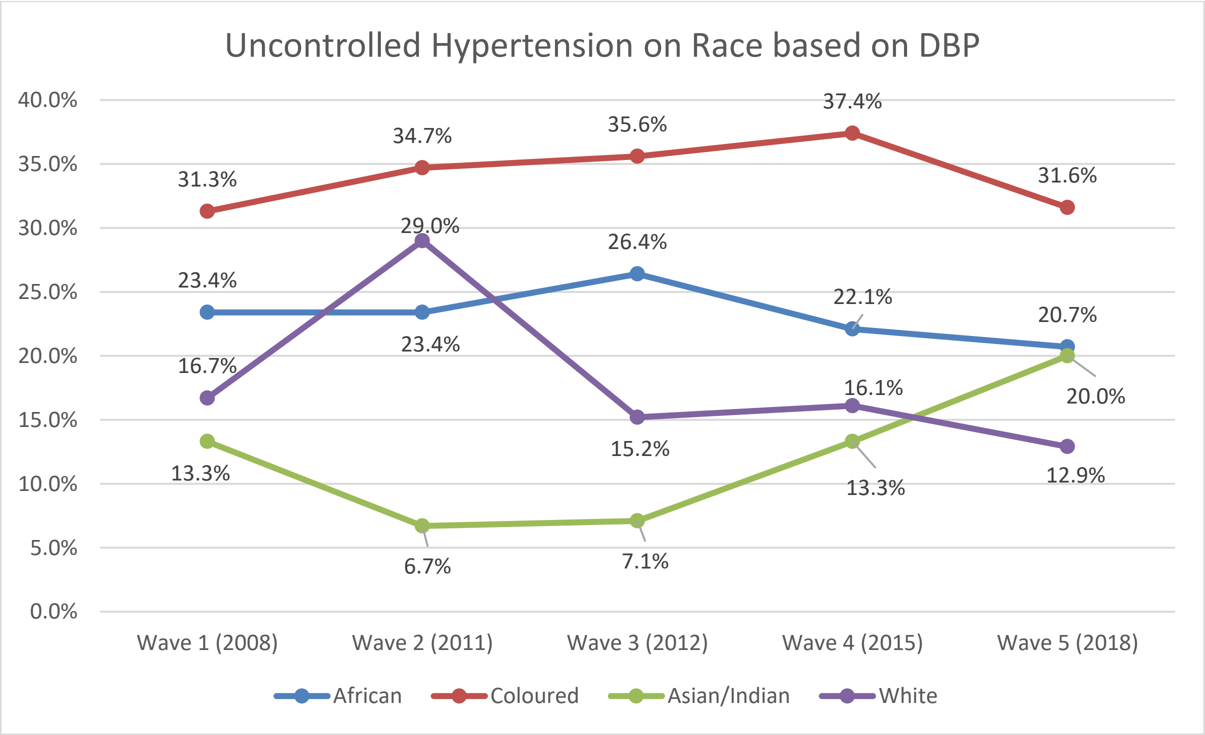

Figure S10: Uncontrolled hypertension on Race based on DBP

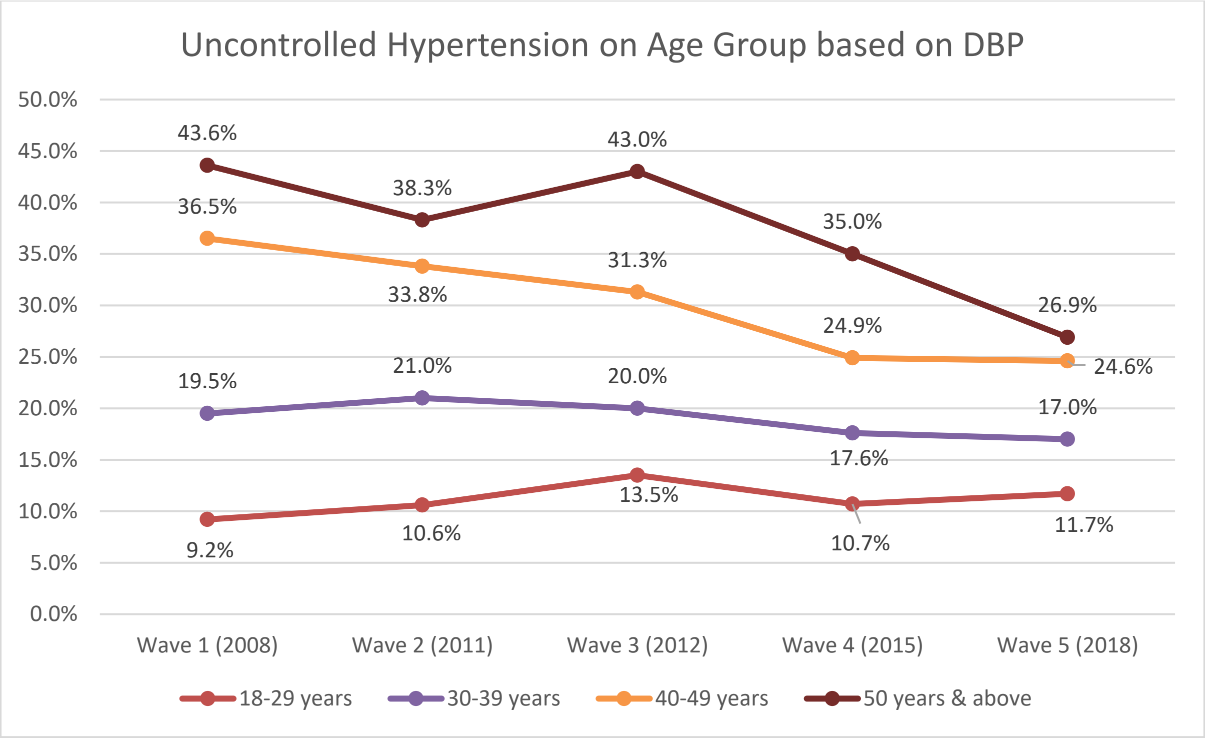

Figure S11: Uncontrolled hypertension on Age Group based on DBP

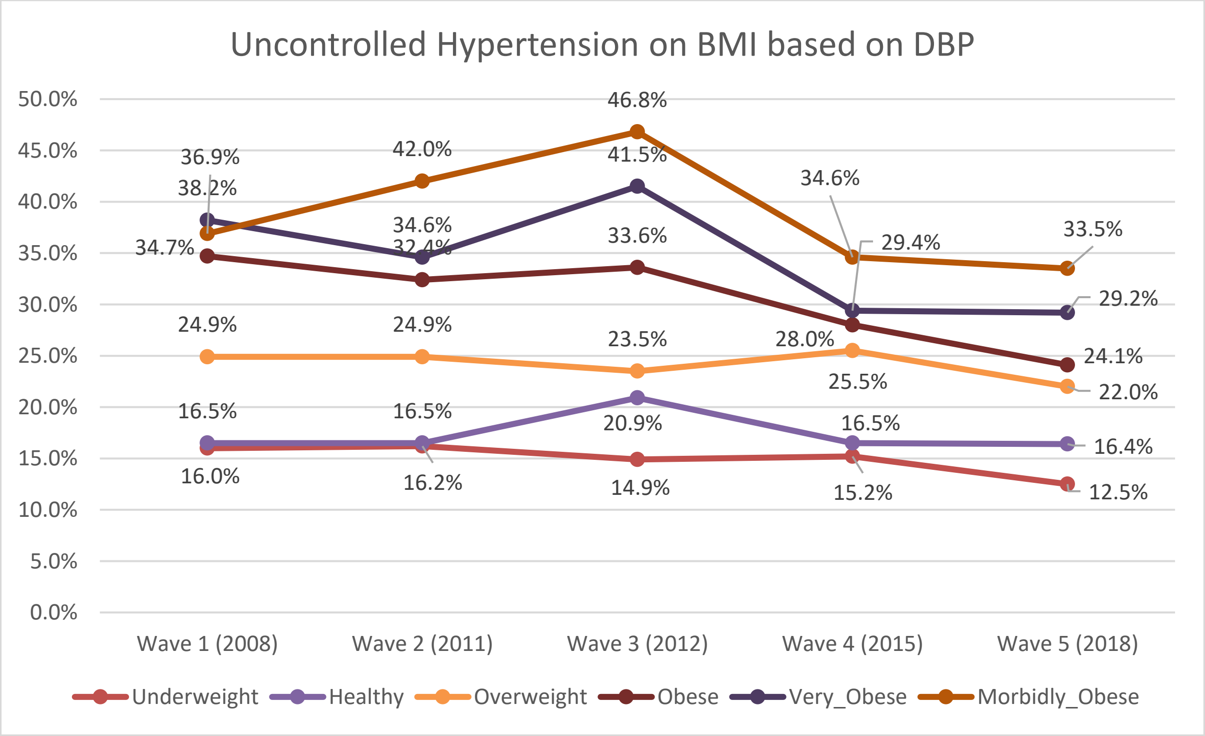

Figure S12: Uncontrolled hypertension on BMI based on DBP

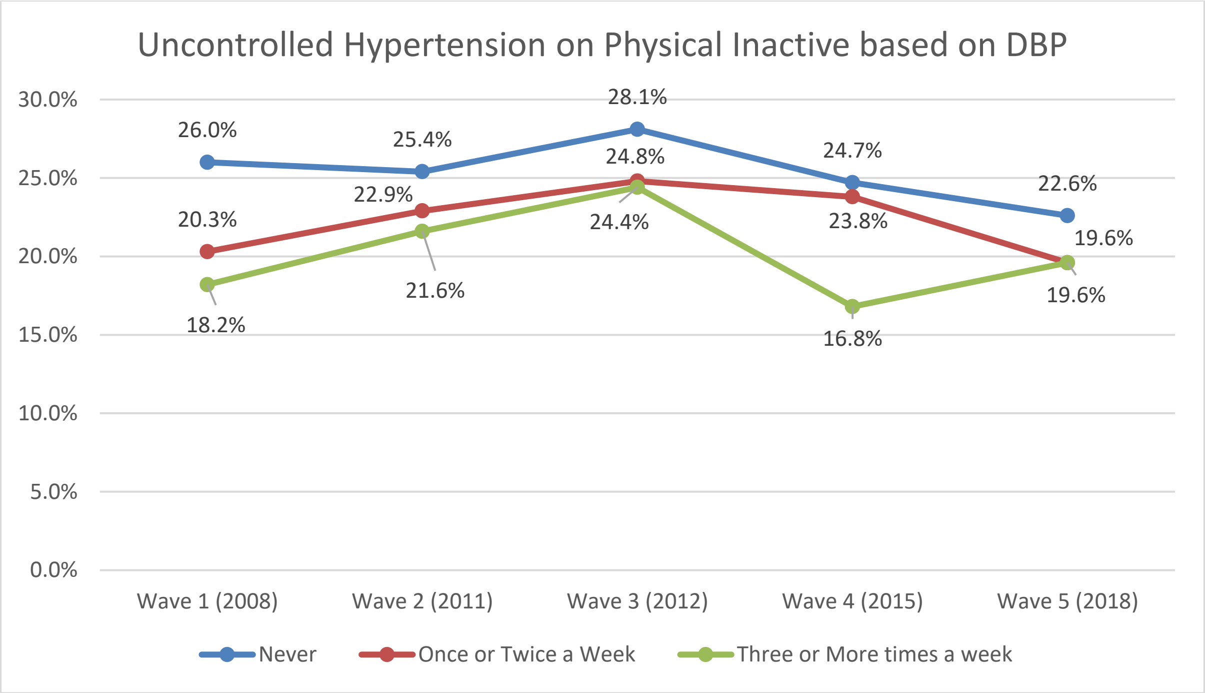

Figure S13: Uncontrolled hypertension on Physical Inactive based on DBP

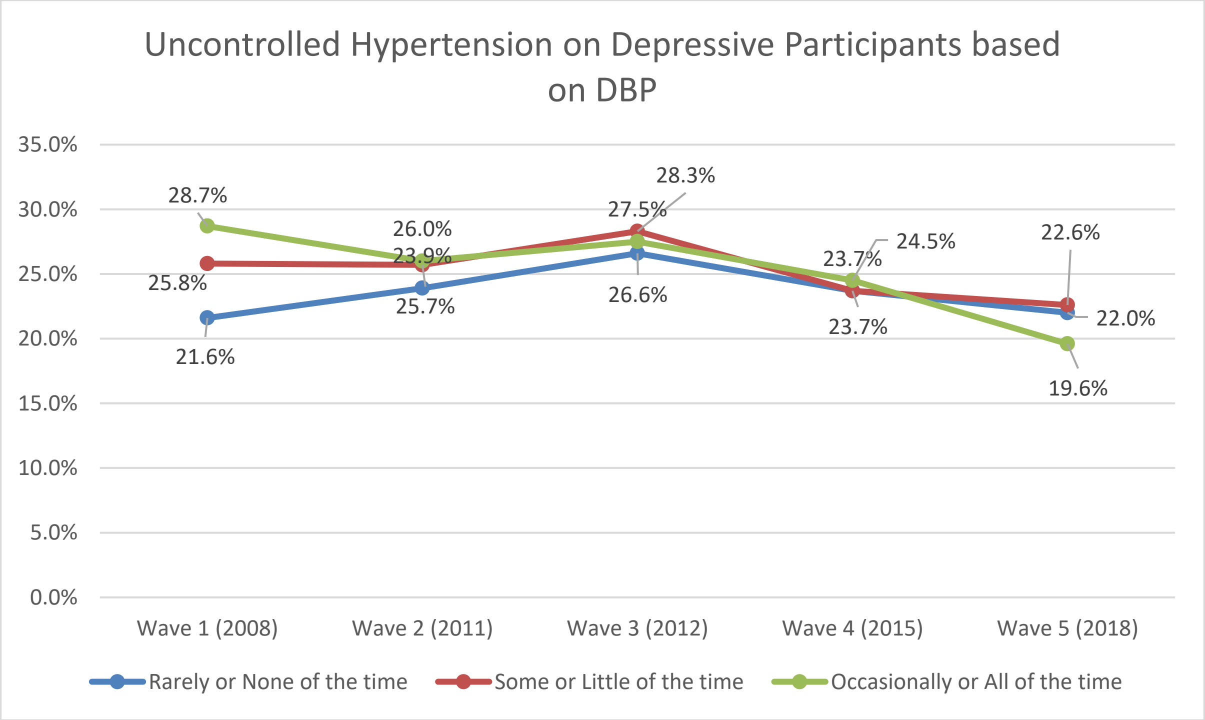

Figure S14: Uncontrolled hypertension on Depressive Participants based on DBP

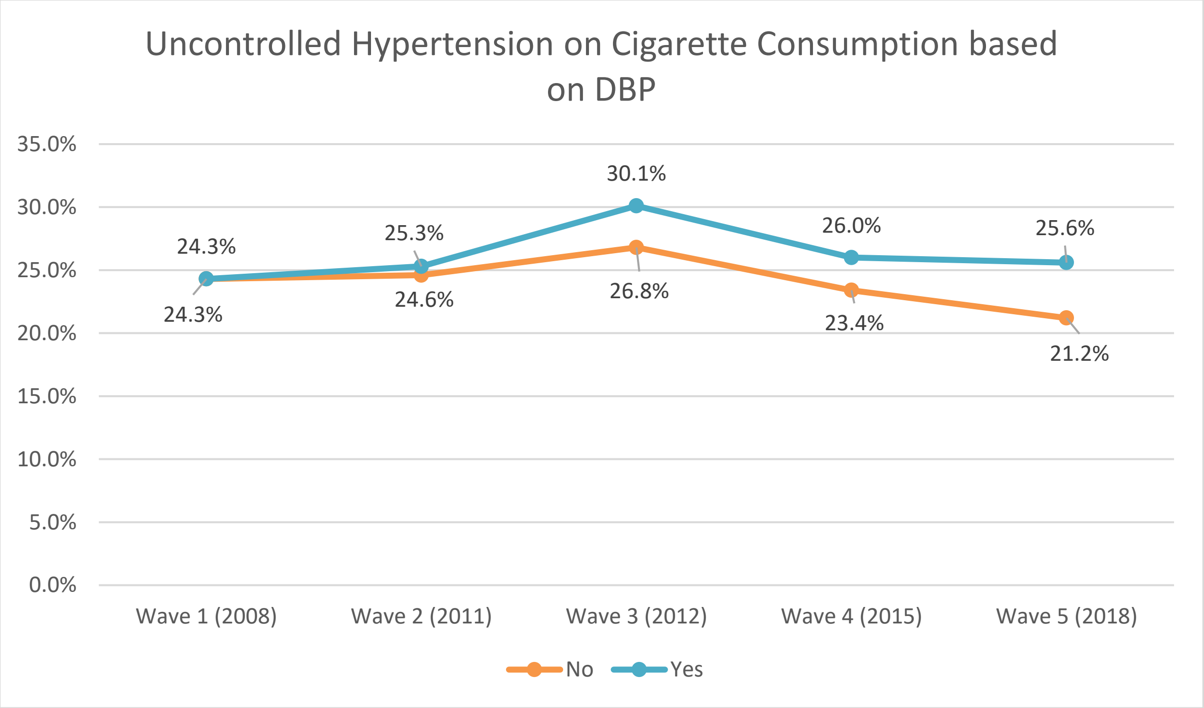

Figure S15: Uncontrolled hypertension on Cigarette Consumption based on DBP

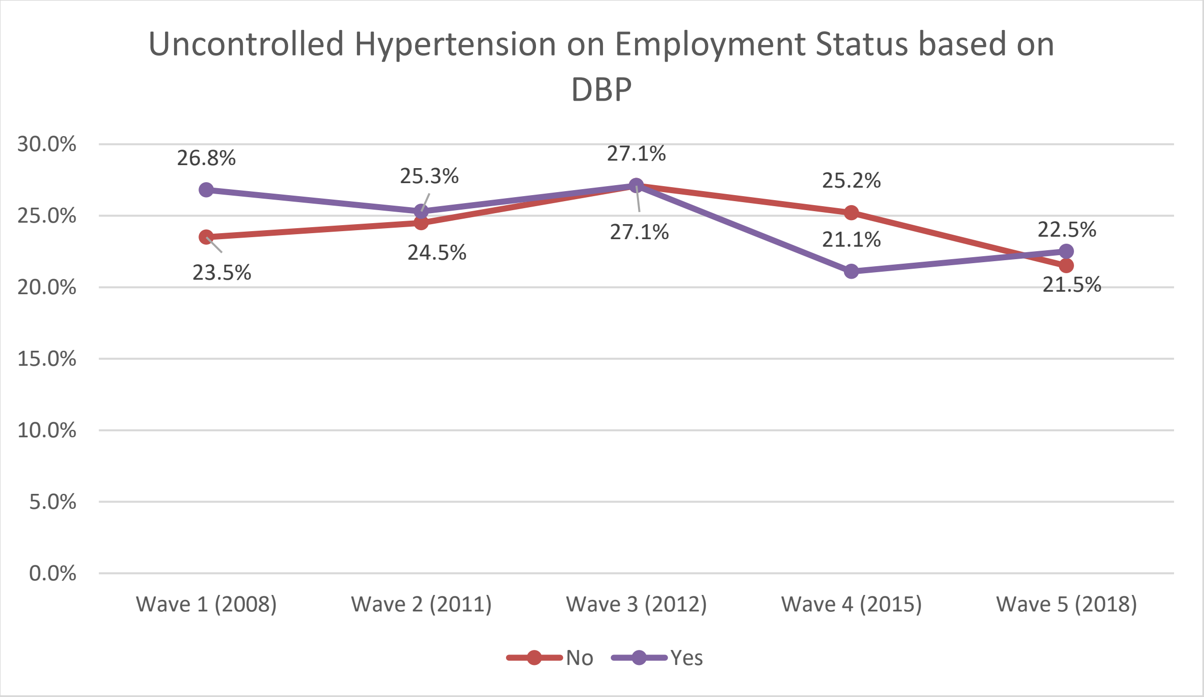

Figure S16: Uncontrolled hypertension on Employment Status based on DBP
